# Neuroscientific Techniques Applied to Forensic Investigation: Innovative Tools for the Assessment of Deception, Recidivism and Memory

**DOI:** 10.1192/j.eurpsy.2026.11286

**Published:** 2026-07-31

**Authors:** G. M. Festa, I. S. Lancia

**Affiliations:** 1 Pontifical Faculty of Educational Sciences «AUXILIUM»; 2 Interdisciplinary Institute of Advanced Clinical Training «IACT», Rome, Italy

## Abstract

**Introduction:**

In recent years, neuroscientific techniques have gained increasing attention in the forensic field due to their potential to provide new insights into brain functioning in relation to behavior relevant to judicial contexts. Technologies such as Functional Magnetic Resonance Imaging (fMRI), Event-Related Potentials (ERP), Brain Fingerprinting, and Behavioral Biometrics represent promising tools for integrating the analysis of human behavior in investigative and legal processes. For further discussion, see: Festa, G. M., Lancia, I. S. (2025). *Neuroscientific and Biometric Methodologies in Forensic Contexts: A Critical Review.* *Psychology and Behavioral Sciences*, 14(2), 43–51.

**Objectives:**

This paper aims to explore the applicability of selected innovative neuroscientific techniques in three specific areas of forensic psychology:

Detection of deception;

Identification of recidivistic behaviors;

Retrieval of memory traces related to specific events.

**Methods:**

A review of recent scientific literature was conducted regarding the use of the following technologies:

fMRI: for detecting brain activity during complex cognitive tasks;

ERP: for the temporal analysis of neural responses to relevant stimuli;

Brain Fingerprinting: for measuring the P300 response in implicit recognition tasks;

Behavioral Biometrics: for analyzing psychophysiological and behavioral parameters such as language, posture, and microexpressions.

**Results:**

Current evidence suggests that these techniques may significantly contribute to the identification of latent information, the analysis of the credibility of certain statements, and the reconstruction of memory content. Nonetheless, several methodological issues emerge, including low replicability, challenges in controlling confounding variables, and ethical and legal concerns related to the use of cerebral and biobehavioral data in judicial contexts.

**Conclusions:**

Forensic neuroscience is a rapidly evolving field with significant practical potential, although still constrained by scientific and ethical limitations. The techniques reviewed should be regarded as complementary, rather than substitutive, tools in relation to traditional evidence. Further interdisciplinary research and the development of standardized protocols are essential to ensure the reliability and legal admissibility of these tools in forensic settings.

**Disclosure of Interest:**

None Declared

